# The Family-Check-Up® Autism Implementation Research (FAIR) Study: protocol for a study evaluating the effectiveness and implementation of a family-centered intervention within a Canadian autism service setting

**DOI:** 10.3389/fpubh.2023.1309154

**Published:** 2024-01-16

**Authors:** Teresa Bennett, Irene Drmic, Julie Gross, Marc Jambon, Melissa Kimber, Anat Zaidman-Zait, K. Andrews, Julia Frei, E. Duku, Stelios Georgiades, Andrea Gonzalez, Magdalena Janus, E. Lipman, Paulo Pires, Heather Prime, Caroline Roncadin, Mackenzie Salt, Rebecca Shine

**Affiliations:** ^1^Department of Psychiatry and Behavioral Neurosciences, Offord Centre for Child Studies, McMaster University/McMaster Children’s Hospital, Hamilton, ON, Canada; ^2^Ron Joyce Children’s Health Centre Hamilton Health Sciences/McMaster Children’s Hospital, Hamilton, ON, Canada; ^3^Department of Psychology, Wilfrid Laurier University, Waterloo, ON, Canada; ^4^Tel Aviv University, Tel Aviv, Israel; ^5^Department of Psychology, York University, Toronto, ON, Canada

**Keywords:** autism, family-centered care, parenting, Family Check-Up, implementation, family intervention, caregiver intervention

## Abstract

**Introduction:**

Prevalence rates of emotional and behavior problems (EBP) in autistic children and youth are high (40–70%), and often cause severe and chronic impairment. Furthermore, autistic children are also more likely to experience family “social-ecological” adversity compared to neurotypically developing peers, including social isolation, child maltreatment, caregiver mental illness, and socioeconomic risk. These family stressors increase the risk of co-occurring EBP among autistic children and can often impede access to evidence-based care, thus amplifying long-term health inequities for autistic children and their caregivers. In the current autism services landscape, there are few scalable, evidence-based programs that adequately address these needs. The *Family Check-Up (FCU®)* is a brief, strength-based, and tailored family-centered intervention that supports positive parenting and explicitly assesses the social determinants of child and family mental health within an ecological framework. Studies have demonstrated long-term positive child and caregiver outcomes in other populations, but the FCU® has not been evaluated in families of autistic children and youth. Therefore, we aimed to evaluate FCU® implementation within an established, publicly funded Autism Program in Ontario, Canada, with delivery by autism therapists, to demonstrate sustainable effectiveness within real-world settings.

**Methods:**

In this study, we outline the protocol for a hybrid implementation-effectiveness approach with two key components: (1) A parallel-arm randomized controlled trial of *N* = 80 autistic children/youth (ages 6–17 years) and high levels of EBP and their caregivers. Primary and secondary outcomes include child EBP, and caregiver well-being and parenting. (2) A mixed methods implementation study, to describe facilitators and barriers to implementation of the FCU® within an autism service setting.

**Discussion:**

Scalable, ecologically focused family-centered interventions offer promise as key components of a public health framework aimed at reducing mental health inequities among autistic children, youth, and their caregivers. Results of this study will inform further program refinement and scale-up.

## 1 Introduction

“*Growing up in Canada is like a race. I do not mind if my children are in a race as long as the race is fair*” –Dr. David (Dan) R. Offord, Child Psychiatrist, 1934–2004.

Engaged, peaceful and well-supported participation of children and youth with disabilities in the major school, home, and leisure domains of their lives is a fundamental determinant of mental health. It is also cornerstone of equity for any society seeking to “make the race fair” for children who fall behind too often. For all children, including those with disability, this includes recognizing both their unmet needs and the assets they bring to their communities, reducing chronic sources of stress, and ensuring that caregivers (e.g., parents) have the resources they need to support their children’s healthy development and their family’s well-being.

Autistic children and youth who also experience co-occurring emotional and behavior problems (EBP) comprise a group that is at particularly high risk of exclusion from meaningful daily social participation in schools and communities. Autism spectrum disorder (ASD) has an early-onset, highly heritable neurodevelopmental profile characterized by core challenges in social communication as well as rigid, restrictive or repetitive behavior and interests and/or sensory sensitivity with 31–55% experiencing co-occurring intellectual disability (1, 2). Up to 70% struggle with problems such as anxiety, hyperactivity, mood difficulties, and challenging behaviors (3). EBP signal increased risk of chronic impairment that cascades across multiple settings (4, 5) and developmental stages from early childhood to later adulthood for autistic people and their families (6–8).

There is growing evidence that the health and development of autistic children and youth are meaningfully influenced by their “developmental ecology,” i.e., their lived environments, which are in turn influenced by more distal social contexts (e.g., neighborhood cohesion, societal income equality, and social welfare policies) (5, 9–12). Autistic children and their families are also more likely to experience ecological adversity, including caregiver marital strain (6), depression (7), stress (3, 8), child experience of bullying (9), and under-involvement in protective social experiences (e.g., friendships, recreational activities) (10, 11). According to Developmental-Ecological models, the daily interactions, routines, and relationships experienced within their family units are most closely and often reciprocally influential (13, 14). Caregivers (e.g., parents) influence emotional and behavioral adjustment among autistic children, especially during key developmental periods, including transitions to school-aged, adolescence, and young adulthood years (5, 10, 15).

Recent longitudinal studies provide compelling evidence supporting the need to integrate an ecological approach into autism and mental health services. One example includes the Canadian Pathways in ASD Study, which is a longitudinal cohort study following over 400 preschoolers from time of ASD diagnosis to late adolescence. Across multiple separate “Pathways” peer-reviewed publications that have examined this data, family socioeconomic status (SES) and relationships, social supports, caregiver depression, stress, and coping have all been linked to later child EBP (5, 12, 16, 17). Furthermore, researchers found that distinct profiles of child and family risk and protective factors may identify families in need of targeted or more intensive support to prevent or diminish child (and family) mental health and developmental risk. Caregiver stress at time of diagnosis was specifically associated with child EBP, family dysfunction and specific caregiver coping styles, and predicted persistent caregiver stress (16). However, caregiver-reported social supports appeared to be protective. Furthermore, children whose families experienced the greatest degree of adversity (e.g., lowest access to social resources and informal supports, high SES risk, and disengaged caregiver emotional coping style) had significantly more impaired behavioral and adaptive functioning outcomes 2 years later. These caregivers also experienced highest levels of personal distress (18).

Supporting parenting and positive family relationships is thus a promising child mental health prevention and intervention approach. Correspondingly, parenting programs have demonstrated benefits among families of autistic children (19, 20), however, provider training and uptake of such programs is low (21). Furthermore, research in populations of both autistic and non-autistic children indicates that more severe child behavior problems and family-level strain (e.g., caregiver depression, low income) often pose barriers to engagement in, and benefit from, such programs, which are typically offered within group modalities, or without an initial assessment of needs (22–24). In contrast, more flexible, tailored, 1:1 approaches may retain and benefit highest-needs families most strongly (25, 26) and thus, may be an important option within a suite of services aimed at supporting autistic children.

Collectively, evidence from longitudinal research indicates that comprehensive mental health interventions for autistic children and youth should seek to decrease barriers to care, systematically assess known modifiable, contextual risk and protective factors for EBP and engage caregivers in a meaningful way as agents of positive change and mental health support for their child. A strength-based approach is particularly essential given higher than average rates of stress and depression among caregivers of autistic children and the harmful psychiatric history of blaming mothers as “causes” of their child’s autism (27). However, to our knowledge, assessment-driven, tailored, family-centered models that assess and act upon ecological risk and protective factors related to child EBP (e.g., caregiver well-being, social supports, family cohesion, and parenting) have yet to be tested among families of autistic children. Furthermore, this is a lifespan problem: social-ecological disparities commonly persist into adulthood, in ways that include social isolation and underemployment (28, 29). Therefore, engaging families of autistic children and youth as early as possible across childhood and adolescence is essential.

### 1.1 The Family Check-Up®

The FCU® (federally registered trademark, University of Oregon) is a brief, evidence-based, trans-diagnostic intervention developed to decrease childhood EBP and related impairment (22) by (1) assessing known ecological (child, family, and contextual) risk and protective factors, (2) engaging caregivers in a strength-based, motivational feedback session and plan to enhance positive parenting and family management skills, and (3) connecting participants to a tailored suite of child and family supports within agencies and communities, which may include a tailored, evidence-based package of parenting sessions [“Everyday Parenting Curriculum (EDP)” (23)]. American and European studies of non-autistic children and youth indicate that the FCU® has robust and sustained benefits for child, youth, and young adult emotional and behavioral well-being and related functioning, caregiver mental health, and family connectedness to services (28–31). However, this intervention has not been evaluated within the context of an Autism Service as delivered by primary autism behavioral service providers.

### 1.2 Initial feasibility and acceptability work

The current study builds on an initial mixed methods acceptability study of the FCU® as provided to families of autistic children and youth aged 6–17 years old who provided qualitative input on their experience of the intervention and related research measurement battery. A Master’s-level social worker with extensive mental health and family therapy experience was trained and credentialed by FCU® developers to deliver the model to 19 families of autistic children and youth without co-occurring intellectual disability, referred to the program by mental health or developmental pediatrics providers because of significant emotional and/or behavioral problems (e.g., emotional dysregulation, dysphoria, and aggression). Caregivers found the FCU® to be relevant to their families’ needs, particularly the emphasis on the “whole family” including relationships between caregivers and their mental health, and the opportunity to engage in a shared feedback session with older children and youth. Several participants noted that a strength-based approach to parenting was particularly important—that they had previously often felt that they must be doing things “wrong.” They recommended expanding the program to include families of children and youth with lower levels of language and intellectual capacity and to shorten the research measurement battery. This work demonstrated that the FCU® can be delivered with fidelity by Master’s trained mental health professionals to families of autistic children; however, it is unclear whether it can be feasibly delivered by the class of therapists employed in autism programming in Ontario, who often have undergraduate-level credentials and training in behavioral [e.g., applied behavioral analysis (ABA)], as compared to psychotherapeutic or caregiver training, interventions.

### 1.3 Study objectives

The current study was designed by a team of clinicians, researchers, and developmental and mental health service administrators with expertise in autism, EBP, and intervention science. Our primary objective was to obtain preliminary estimates of the effectiveness of the Family Check-Up (FCU®) compared to treatment as usual in an Ontario (Canada) sample of 80 families of autistic children and youth aged 6–17 years old who are registered to receive care within a regional autism service and evaluate the feasibility of implementation within this setting. This work aligned with shifts in an Ontario health policy context calling for increased spending on family and child mental health supports, in response to an expert clinical and community stakeholder report (32).

### 1.4 Family engagement

We have engaged a Family Advisory Committee to advise on the conduct of the study. The seven-person committee represents families of autistic children with ASD who have participated in the FCU® feasibility study: they provide feedback on exploratory effectiveness measures and caregiver and youth interview guides, advise on recruitment and referral throughout the trial, and will support interpretation of study results and knowledge translation. Members co-develop terms of reference, meet twice/year and receive a stipend. For the proposed study, in response to caregiver input through the feasibility study and advisory groups, we have: adopted more broad and pragmatic inclusion/exclusion criteria, dropped adaptive functioning measures as research outcomes (too burdensome), included FCU® assessments addressing sibling relationships, screen time and online monitoring (identified by families as important indicators), changed wording of some task instructions and included an annual “booster” FCU® at 12 months. Furthermore, our choice of unblinded caregiver reports of child EBP as primary outcome was validated by feasibility study participants’ reports that brief caregiver-child interaction tasks were very helpful clinical tools but not indicative of the full range of their child’s emotional and behavioral challenges over time (i.e., low ecological validity).

## 2 Methods and analysis

### 2.1 Study design

The step-wise progression of evidence-based practice from efficacy to effectiveness research, and then to eventual implementation into community practice, has traditionally encompassed a lengthy undertaking that has often resulted in poor intervention effects in real-world settings (24, 25). An implementation science approach seeks to shorten this research-practice gap by considering and evaluating outcomes that genuinely reflect real-world settings and concerns (24, 25). We will employ a Type 1 hybrid implementation-effectiveness approach studying the FCU®. This entails evaluating the program’s effects on the emotional well-being and functioning of autistic children and their caregivers (e.g., parents) as delivered by autism therapists trained in the FCU® model using a proof-of-principle randomized controlled trial (RCT) design, integrated with a mixed methods study focused on concurrently and explicitly evaluating facilitators and barriers to the implementation of the clinical intervention within an autism service setting (33). This design is particularly relevant when there is strong face validity for implementing an intervention in a new setting and/or population, indirect evidence of efficacy (e.g., evidence in other populations), and strong impetus to effect systems-level change.

The current study therefore includes two blended components:

#### 2.1.1 Effectiveness

The effectiveness study design is a parallel-group effectiveness randomized controlled trial (RCT) within an Autism Service located within a regional tertiary healthcare center serving a large city and surrounding small-urban and rural areas in Ontario, Canada. A sample of 80 children aged 6–17 years who are functionally speaking (or, “functionally verbal”) with clinically confirmed diagnoses of ASD and high levels of EBP and their families will be enrolled and randomized into either the FCU® or treatment as usual (TAU) with outcome assessments at 0, 3, 6, 9, and 12 months.

We will estimate the effectiveness of participation in the FCU® (+ up to 6 months of optional EDP sessions) vs. Treatment as Usual (TAU) by families of autistic children and youth aged 6–17 years in (a) decreasing child EBP (primary outcome), (b) decreasing caregiver depression, parenting stress, and (c) increasing positive parenting practice. (d) We will describe qualitative and quantitative differences in child and caregiver outcomes and connectedness to child and family services between intervention arms, and between FCU® participants classified as responders vs. non-responders.

Primary and secondary outcomes will be measured at 6 months, with follow-up visits at 9 and 12 months to determine if these effects fade out or are sustained.

#### 2.1.2 Implementation

Concurrent with the effectiveness evaluation, we will conduct a mixed-methods study aimed at evaluating delivery of the model and describing contextual factors and barriers to FCU® implementation and sustainability within a regional autism service setting. This work will be informed by the Exploration, Preparation, Implementation, Sustainment (EPIS) framework (34), which focuses on evidence-based practice implementation in publicly funded services, and the FCU® Implementation Framework (35), which emphasizes the inner context and FCU®-specific facilitators and barriers at each EPIS stage (36). Specifically, implementation aims are to:

1. Evaluate metrics related to the adoption, implementation, and sustainable delivery of the FCU® within the urban outpatient hospital-based Autism Program and characterize implementation facilitators and barriers using a mixed-methods approach.
2. Describe autism therapists’ experience of FCU® training, supervision and delivery, and measure sustained competence and fidelity FCU® model delivery.
3. Describe leadership impressions/experiences of providing the FCU® within the wider Autism Services setting and obtain key administrative metrics related to clinical delivery.
4. Obtain caregiver and youth impressions of participating in the FCU® as provided within an Autism Program setting.
5. Describe the processes and effectiveness of outreach, screening, and referral approaches to inform future implementation efforts.

#### 2.1.3 Sample and recruitment

The study sample consists of 80 children/youth with ASD and high levels of EBP and their caregiver(s).

##### 2.1.3.1 Inclusion criteria

1. Child 6–17 years of age.
2. Confirmed diagnosis of ASD.
3. Enrollment in the Ontario Autism Program (OAP).
4. Minimum developmental age of 2 years.
5. Elevated EBP as determined by high or very high scores on the emotional problems (≥ 5), hyperactivity (≥ 8), and/or conduct problems (≥ 4) scales of the Strengths and Difficulties Questionnaire (SDQ) (37) OR a score ≥ 12 on the irritability subscale of the Aberrant Behavior Checklist (ABC) (38).
6. Residing with the same caregiver for at least 5 days/week OR every other week for the past 2 months and the foreseeable future.

##### 2.1.3.2 Exclusion criteria

1. Caregiver with insufficient knowledge of English to complete questionnaires.
2. Current enrollment in another intervention study.
3. Active significant safeguarding concerns (e.g., child with severe acute self-harm or aggression requiring hospitalization; acute caregiver suicidality; and medical fragility).
4. Prior participation in the FCU® in another setting or study.

Recruitment settings include: referrals from family service coordinators who support service navigation within the regional autism program, ASD diagnostic hubs, school boards, community organizations, and healthcare providers. Families may also self-refer or be referred by other research study staff (provided they complete a consent to contact so their information can be shared with research staff).

#### 2.1.4 Screening

Interested caregivers will complete a 20–30 min telephone or in-person screening interview with research staff to hear about the study and assess inclusion/exclusion criteria. Families will be asked some basic questions to assess eligibility (e.g., child age, child’s primary residence and caregivers, participation in other studies, child’s language abilities, and child’s developmental age). Child EBP will be assessed as described above.

The FCU® will be provided within the regional Autism service by government-funded clinicians, therefore participating families must be registered with the provincial OAP. Interested families who are unregistered will be connected to service navigation to facilitate this process.

#### 2.1.5 Randomization and blinding

Randomization will occur following the baseline visit to reduce risk of differential attrition. Participants will be randomly assigned to one of two conditions (FCU® or Treatment as Usual) using an internet-based randomization service; https://www.randomize.net/. Randomization will be stratified by child chronological age (6–10 and 11–17 years) and by presence/absence of co-occurring intellectual disability. Participants will be informed of their treatment arm status by the Research Coordinator or another research staff member who will not conduct any follow-up measures.

Participants will be informed of their right to withdraw from the study at any time. Furthermore, the lead principal investigator (LPI) may decide to withdraw a participating family from the study, if required, to mitigate undue risk to caregiver, child, research staff, or FCU® clinician.

Study participants will be withdrawn from the study under the following conditions:

- If it is determined that the caregiver or child has an acute psychiatric crisis (e.g., psychosis) that will interfere with the ability to participate in the study.
- If a caregiver is experiencing an extreme crisis (e.g., related to an abusive or violent relationship) that interferes with the ability to participate in the study.
- If caregiver, child, research staff, or FCU® clinician experience an adverse event that is deemed by the LPI be an unacceptable safety risk.
- Death of child or participating caregiver.
- If participant behaviors or circumstances are deemed to unduly compromise the safety of the treating clinician or research staff (e.g., violence or unsafe behaviors toward research staff or clinician; unsafe conditions in home).
- If the LPI deems it is in the participants’ best interest to discontinue the study treatment.
- Loss of custodial caregiver status, if consent not obtained from replacing custodial caregiver (e.g., other caregiver, child protection service).

### 2.2 Intervention: the Family Check-Up®

**Figure 1:**
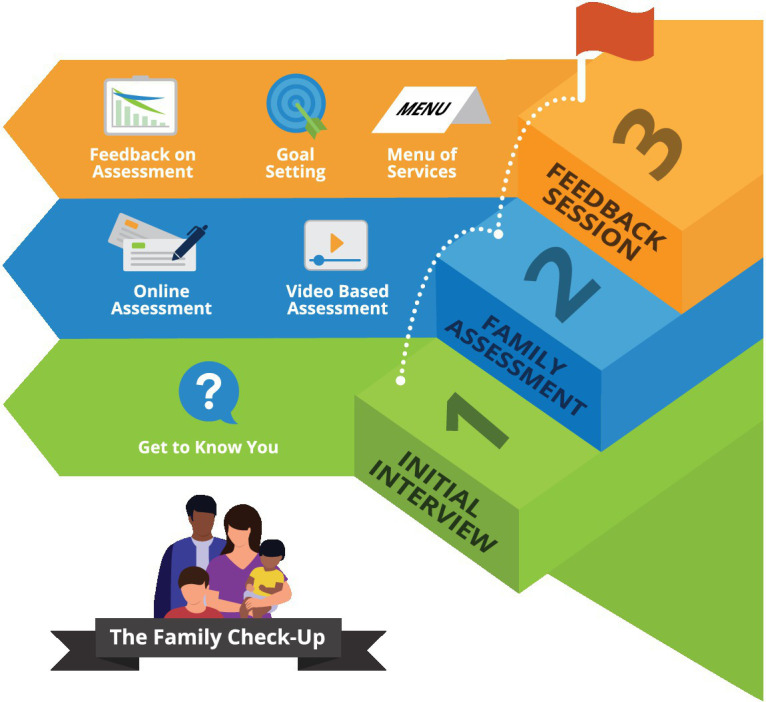
Outline of Family Check-Up® Visits.

The FCU® is a brief “assessment-as-intervention” that engages caregivers in a collaborative process of assessment, reflection, teaching, and goal-setting. The process typically includes three visits (See Figure 1):

#### 2.2.1 “Get to Know You” interview (45 min)

The FCU® clinician introduces the FCU® and engages caregivers in an initial interview aimed at establishing a clinician-client relationship, building rapport, and gathering information about unique child and family strengths and challenges, past successes, and future goals.

#### 2.2.2 Ecological assessment (60 min)

The assessment visit involves questionnaire and observational tasks to assess risk and protective factors across broad domains: family psychosocial context (e.g., SES, supports, caregiver mental health, and partner support), family management (parenting, family warmth and conflict), and child emotional-behavioral well-being, peer relations, and school success. Questionnaires are completed by primary and any additional participating caregivers and children and youth with developmental ages of at least 11 years.

To address the heterogeneity of social-communication, cognitive and language skills across the spectrum of autistic children and youth, observed Family Interaction Tasks (FITs) and their instructions are tailored to the developmental age of the child. Because the aim is to measure caregiver-child interactions as naturalistically as possible, caregivers are instructed to communicate with their child in their typical way and to support their child to complete the tasks or talk with them about a topic.

Caregivers and children developmentally aged 2–5 years engage in a teaching task, engagement in collaborative play, and clean-up. A selection of toys with broad developmental age ranges were chosen to accommodate older youth who may fall within a developmental age of 2–5 years for the purposes of this task. For children aged 6–10 and 11–17 years, tasks are more discussion-based and address child and family strengths, school experience and goals, parental online monitoring, solving a family problem, and planning a fun family activity. Tasks are designed to elicit key domains of parenting behavior shown to be important for emotional and behavioral adjustment across age groups (i.e., relationship-building, positive behavior support, limit-setting and monitoring, and non-reactive parenting). The interactions are videotaped, coded by the clinician according to established FCU® guidelines and incorporated into the feedback with 2–3 clips chosen to highlight child/youth and caregiver strengths and positive interactions, emphasizing examples of effective parenting skills and child/youth response.

1. Collaborative feedback session (60–90 min): The FCU® clinician provides structured feedback to caregivers based on assessment results using motivational interviewing techniques to engage the caregiver in reflection and “change talk (39)”. The discussion is scaffolded by a visual feedback form that integrates questionnaire, interview and video-based data as well as brief, empirically supported rationales about the interdependencies of child adjustment, parenting, and the family context, tailored to individual child and family profiles. The clinician will also incorporate relevant autism-related child strengths and challenges and a parenting lens, emphasizing evidence-based transdiagnostic positive parenting behavior that support child and youth self-regulation. Strength-based video clips highlighting skillful parenting behaviors and positive parent–child interactions are chosen to optimize caregiver engagement, self-efficacy and motivation. The clinician supports the caregiver to outline goals and collaboratively design a tailored menu of services, with service navigation and advocacy support as needed. Examples of “menu items” may include caregiver engagement in EDP sessions focused on one or more parenting behavior domains collaboratively identified as an area of need, connection to child and youth mental health programs, caregiver support to connect to own mental health services, child recreation programs, housing, or other funding application support.

#### 2.2.3 Everyday Parenting (EDP) curriculum

The FCU® clinician and caregiver (s) may decide upon a suite of optional 1:1 EDP sessions, the number and content of which are carefully tailored to parenting strengths, challenges, and goals established during the collaborative FCU® feedback session. The EDP (41) is based on a Social Interaction Learning (41, 42) model of parenting; it supports caregivers to become mindful of interaction patterns with their children (both positive and negative) and to strengthen positive caregiver-child relationships and parenting skills to scaffold child self-regulation. Sessions are provided weekly to biweekly in-person or by Zoom for up to 6 months, an arbitrary cut-off chosen for clinical resource and study timeline purposes, the acceptability of which will be re-evaluated upon study completion.

#### 2.2.4 Intervention evidence

The FCU® was developed by Dr. T. Dishion and colleagues in response to decades of research demonstrating how family ecology shapes child mental health risk and resilience (29, 40), and unmet needs for prevention and interventions that effectively engage parents and caregivers living in stressful circumstances. It has been adapted to include families of children from infancy age to young adulthood, and has demonstrated sustained (42), reliable and robust positive effects on multi-informant reports of child, adolescent, and young adult outcomes that are highly relevant to ASD, including direct and indirect effects on: emotional self-regulation (43), disruptive behavior (44), extra-curricular involvement (35) and academic achievement (45), depressive symptoms (46), suicidality (47), family connectedness to service (30), and caregiver mental health (44). The program has demonstrated effective delivery within homes (29), clinics (48), and schools (49).

### 2.3 Study visit schedule

**Figure 2:**
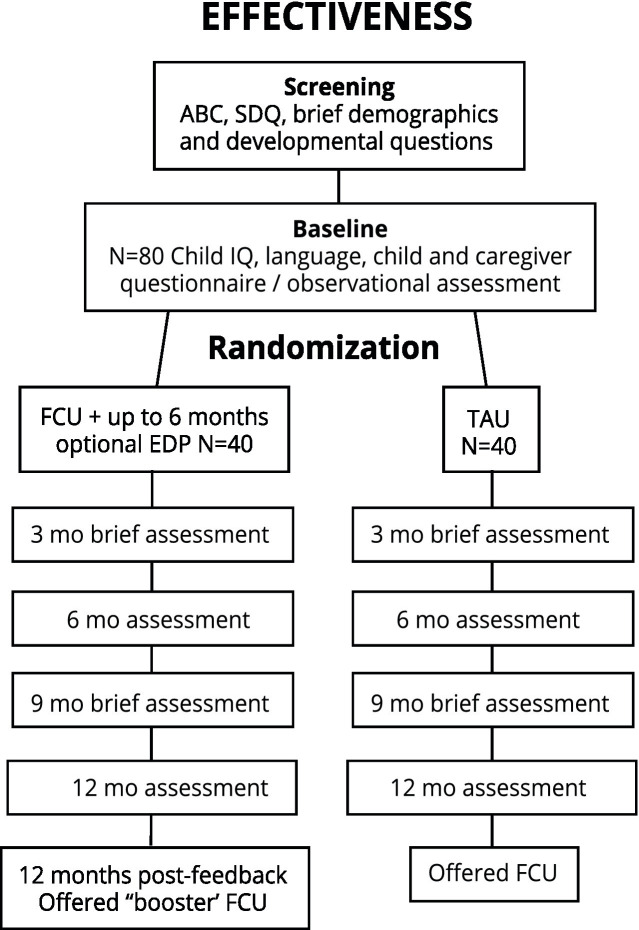
Study flow, effectiveness randomized controlled trial arm. ABC, Aberrant behavior checklist; FCU, Family Check-Up; EDP, Everyday parenting curriculum; SDQ, Strength and difficulties questionnaire.

See Figure 2. This study will implement two key changes from the FCU® format as typically delivered in clinical settings. First, the order of the first and second FCU® visits will be reversed, so that the assessment is unbiased by whether participating caregivers anticipate receiving the FCU® intervention or not. That is, the multimodal assessment will be delivered at the baseline visit prior to randomization (typically the assessment occurs during the second visit of the FCU®). Second, the baseline assessment will be conducted by research staff rather than FCU® clinicians. These changes are standard in FCU® research (29).

#### 2.3.1 Baseline assessment (3.0 h)

The baseline visit will be conducted in clinic. Families will be engaged in a multi-modal assessment including questionnaires and activities. Caregivers and children (developmental age 11+ years) will complete questionnaires that cover primary, secondary, and exploratory outcomes. Brief child language and IQ tests will be administered. In addition, the family will be asked to do FITs, which will be video-recorded for later coding.

Families randomized to the FCU® arm will be informed that an FCU® clinician will contact them shortly for participation in the FCU®. Families randomized to the TAU arm will be connected to a Family Service Coordinator in the Autism Program, who can direct them to appropriate services that are freely or available as a fee-for-service option. For ethical reasons, families randomized to the TAU arm will have the option to receive the FCU® upon study completion.

#### 2.3.2 FCU® visits (FCU® arm only)

All FCU® visits will be conducted by a clinician and will take place in clinic or virtually (dependent on the family’s preference). The Research Coordinator will provide the baseline assessment data, including responses to questionnaires and videos of the family interaction tasks, to the clinician who will then schedule the initial interview with the caregiver.

##### 2.3.2.1 “Get to Know You” initial interview visit (30–45 min)

The purpose of this initial visit is to describe the FCU® process, build rapport, and obtain preliminary information about the child and family.

##### 2.3.2.2 Feedback visit (60–90 min)

As per the FCU® protocol, the FCU® clinician will engage the caregivers (s) in a feedback session where assessment results are reviewed using a motivational interviewing framework. Youth aged 11 years and older who contribute questionnaire data are invited to receive a separate feedback session with the option of then engaging in a shared feedback session with their parent (s)/caregivers after review of their data and goals. Sensitive caregiver information (e.g., self-reported mental health, parenting) are shared individually with caregivers only and youth choose which aspects of their self-reports they would like to share. The feedback visit concludes with collaborative goal-setting to develop a menu of services that maps explicitly onto family needs. These may include EDP sessions, individual child treatment, connection to parent mental health services, and/or direction to community supports or recreation programs.

##### 2.3.2.3 Everyday Parenting curriculum (60 min—optional)

If appropriate, families may access a suite of optional EDP sessions that are tailored in content and number to their feedback. The feedback and EDP sessions (if applicable) must be completed prior to the 6 months assessment.

#### 2.3.3 3-month assessment (30 min)

This is a brief visit that involves an interview and questionnaires to measure primary and secondary outcomes. It can take place in the family’s home, clinic, a community location, or over the phone with questionnaire completion through emailed links.

#### 2.3.4 6-month assessment (1.5–2 h)

The 6-month visit is a repeat of the baseline assessment, with the exception of child IQ and language assessments.

#### 2.3.5 9-month assessment (30 min)

The 9-month visit involves primary and secondary questionnaire outcomes.

#### 2.3.6 12-month assessment (1.5–2 h)

The 12-month visit is a repeat of the 6-month assessment.

#### 2.3.7 Post-study visits

According to the health maintenance model, annual “check-ups” help maintain gains, bolster skills, and think ahead to the child’s next developmental period. Therefore, families in the FCU® group will be offered a “booster” FCU® visit that incorporates their 12-month assessment data. Families in the TAU group will connected to an FCU® clinician in order to receive the FCU®.

### 2.4 Measures

#### 2.4.1 Screening measures

Families will be asked for basic demographic information to determine eligibility (e.g., child date of birth, child’s primary residence and caregivers). To determine if child has a developmental age of at least 2 years, caregivers will be administered item #41 from the Autism Diagnostic Interview (ADI-R) which assesses how well the child uses language to communicate. They will also be asked whether the child can follow simple two-step commands and if they use any alternative communication devices. In addition, caregivers will be asked questions about the presence or absence of intellectual and learning disabilities.

Child EBP will be assessed as follows:

The *Strengths and Difficulties Questionnaire (SDQ)* (45), a widely used 25-item behavioral screening questionnaire for children ages 2–17 years. The Prosocial Behavior scale showcases strengths, while the remaining four evaluate negative behaviors such as emotional symptoms, conduct problems, hyperactivity/inattention, and peer relationship problems. Satisfactory psychometric properties have been reported (45) and scores from the SDQ and the CBCL have been shown to be highly correlated (48). A child is eligible if the conduct score ≥ 4 OR the hyperactivity score is ≥8 OR the emotional problems score is ≥5.

The irritability subscale of the *Aberrant Behavior Checklist (ABC)* (50), a commonly used measure of child EBP in psychosocial and pharmacological RCTs in autism research. Because we aim to recruit children with elevated irritability, participants were included if the ABC irritability score is ≥12 as per norms developed for autistic children and youth (38).

#### 2.4.2 Effectiveness measures

##### 2.4.2.1 Primary outcome

Child emotional dysregulation will be measured using the Aberrant Behavior Checklist (ABC) Irritability subscale (51) and the Home Situations Questionnaire—Autism Spectrum Disorder (HSQ-ASD; parent report; 0, 3, 6, 9, and 12 months) (52).

##### 2.4.2.2 Secondary outcomes

The Clinical Global Impressions Scale, Improvement (CGI-I) (53) (blinded interview; 0, 3, 6, and 12 months) will assess primary caregiver impressions of improvement/worsening of EBP, ranging from complete absence of EBP (1) to “disastrously worse” (7). Children will be classified as “responders” for analytic and descriptive purposes if they demonstrated a 25% decrease on the irritability scale and a CGI-I score of 1 or 2.

Caregiver well-being will be assessed by measuring caregiver depression, caregiver anxiety, and parenting stress (parent self-report; 0, 3, 6, 9, and 12 months) Depression will be measured through the Center for Epidemiological Studies Depression Scale-Revised (CESD-R) (54), a 20-item scale with strong psychometric properties.

Caregiver anxiety will be measured using the brief Generalized Anxiety Disorder-7 (GAD-7) (55); and parenting stress will be assessed using a brief version of the Parenting Daily Hassles (56) and the Autism Parenting Stress Index (57).

Specific parenting behaviors will be measured using the Parenting Young Children (PARYC) (58) and Positive Affect Index (59) (parent self-report; 0, 6, and 12 months). The Parental Monitoring Scale (PMS) (60) will also be administered for older children [parent and youth (11+) self-report; 0, 6, and 12 months).

Parenting self-efficacy and coping will be measured using the Parent Empowerment and Efficacy Measure (PEEM) (61) and the Brief COPE (62) (parent self-report; 0, 6, and 12 months). Caregiver thoughts and feelings about their child, and their relationship with their child, will be audio-recorded and coded by assessors blinded to intervention status, using the Autism-Specific 5-Minute Speech Sample (blinded coders; 0, 6, and 12 months) (63). Observed parenting behavior will be coded by blinded observers using a modified version of the Coder Impressions Inventory (COIMP) (64).

Connectedness to and use of services will be measured at 0, 6, and 12 months using a modified version of the Service Utilization Questionnaire developed for a previous Canadian Family Check-Up study.

##### 2.4.2.3 Baseline covariates, mediators, and moderators

Child cognitive skills will be measured using the *Stanford-Binet Intelligence Scales, 5th* Edition routing version (65), which can be administered to people ages 2–85 years. The routing version assesses nonverbal fluid reasoning and verbal knowledge and takes about 15 min to administer. Because the FCU® was developed for families with children ages 2–17 years, it was determined that children taking part in the study must have a minimum developmental age of 2 years. Interested families are asked questions during screening to get a sense of their child’s developmental age, which is measured more formally through the Stanford-Binet at the baseline visit. If this testing determines that a child is developmentally below the age of 2, families will not be able to continue in the study. In this case, they will be provided the gift cards for the baseline visit and connected to a Family Service Coordinator in the Autism Program for other program options.

Child language will be measured using the *Oral and Written Language Scales-II (OWLS-II)* (66), a widely used receptive and expressive language assessment suitable for children with ASD. The OWLS-II takes approximately 20 min to administer.

Child autistic symptoms will be measured at baseline using the Social Communication Scale—Current (SCQ-C) (67). Child sleep will be assessed through two questions from the *2014 Ontario Child Health Study (OCHS) Selected Child and Adolescent Psychiatric Interview (CAPI)* (68, 69) that assess child’s average number of hours of sleep and perception of quality of sleep as well as the parent-report *Children’s Sleep Hygiene Scale* (70). Youth participating in the study will complete the self-report *Adolescent Sleep Hygiene Scale* (71).

Caregiver executive function will be measured using the *Executive Skills Questionnaire—Revised (ESQ-R)* at baseline only (72). Caregiver emotional regulation will be measured through the *Difficulties in Emotional Regulation Scale—Short Form (DERS-SF)* (73), an 18-item scale that assesses deficits in regulating emotions.

Parent sleep will be assessed using the Sleep Hygiene Index (74), a 13-item measure that assesses behaviors thought to compromise sleep hygiene. It has good internal consistency, test–retest reliability, and is positively correlated with other sleep measures. Parents will also be asked the average number of hours of sleep they get each night and to rate the quality of their sleep (very good, fairly good, fairly bad, and very bad).

Household chaos will be measured through the *Confusion, Hubbub and Order Scale (CHAOS)* (75), which is a 15-item measure of environmental confusion. Caregiver alcohol use will be assessed through the three-question *Alcohol Use Disorders Identification Test—Consumption* (76) which assesses the potential harmfulness of a person’s alcohol consumption. Caregiver drug use will be assessed using the *Drug Abuse Screening Test (DAST-10*) (77), which has moderate to high levels of reliability and validity.

#### 2.4.3 Implementation measures

##### 2.4.3.1 Quantitative data

Clinical costs will be tracked, including training, clinician hours, supervisor hours, travel, equipment, and administrative costs.

Organizational and clinician readiness for change will be collected from staff, leadership, and clinicians (0, 6, and 12 months) using the *Evidence-Based Practice Attitudes Scale* (78), *Organizational Readiness for Implementing Change* (79), *Acceptability of Intervention Measure* (79), *Feasibility of Intervention Measure* (79), *Intervention Appropriateness Measure* (79), and the *Oldenburg Burnout Inventory* (80).

The *Working Alliance Inventory-Short Revised* (81, 82) is a measure of the therapeutic alliance between a therapist and their client. Is it brief (client version 12 questions, therapist version 10 questions) and has good psychometric properties. It will be administered after the Get to Know You Visit, feedback session, and third and sixth sessions of EDP (if applicable).

Satisfaction with the intervention will be measured by the *FCU® Satisfaction Scale* (administered to caregivers post-FCU®) (48). In addition, “dosage” (the number of FCU® sessions attended by each family) will be tracked.

With written consent from families, clinicians will videorecord their FCU®/EDP sessions to be able to evaluate fidelity to the model. Fidelity will be assessed by the *COACH Fidelity Rating* (83) system created by FCU® developers to assess adherence to key FCU®/EDP components on a 1–9 scale. During each 6-month period of the study, four videotaped sessions/clinician will be randomly drawn to assess average levels of fidelity in early, middle, and sustainability phases.

##### 2.4.3.2 Qualitative data

**Figure 3:**
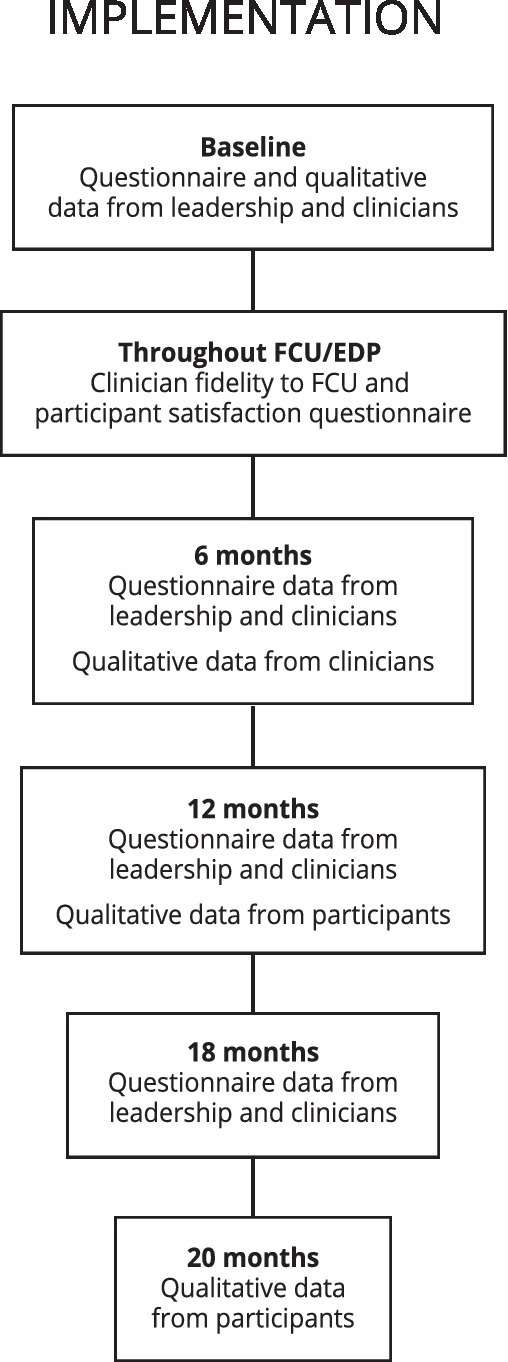
Study flow, implementation design. FCU^®^, Family Check-Up; EDP, Everyday parenting curriculum. ^*^Caregivers and consenting youth invited to qualitative interviews after completing 12-month visit.

See Figure 3. The qualitative component of the implementation study will follow the principles of qualitative description, which is an applied qualitative health research methodology commonly used alongside intervention evaluations. It is well suited to addressing the implementation aims of the present work via its pragmatic emphasis on generating a rich description of the phenomenon of interest by using and staying interpretively close to the words of participants (84, 85). To this end, clinicians and family service coordination staff will be purposefully sampled and invited to join focus groups aimed at understanding contextual barriers and facilitators to FCU® implementation (*N* ~ 10–15; 0, 6, and 18 months). The perspective of leadership will be obtained through individual interviews (*N* ~ 5; 0 and 18 months). Transcribed, coded themes will be fed back iteratively for clinical quality improvement purposes, protecting confidentiality.

Open-ended questions about experience with the FCU® and/or study will be included in 12-month participant questionnaires. In addition, all consenting caregivers and youth (who are capable of consenting) in the FCU® arm will be invited to participate in individual qualitative interviews asking about their experience of family, parenting, and child change vs. stability throughout the intervention, acceptability of the FCU® in this service setting and facilitators/barriers to engagement. When possible, we will briefly interview FCU®-arm participants classified as “non-engagers” (i.e., did not complete the feedback session) about barriers to engagement after their 12-month visits throughout the study.

### 2.5 Analyses

#### 2.5.1 Effectiveness—aim #1

Analyses will be conducted following an intent-to-treat approach. Two-wave latent difference score models will be used to evaluate whether participation in the FCU®, compared to TAU, is associated with greater decreases in child EBP (primary outcome) and caregiver depression and parenting stress from baseline to 6 and 12 months post-baseline, and greater increases in positive parenting from baseline to 6 and 12 months post-baseline. A sensitivity power analysis indicates that an *N* = 80 is powered to detect a significant regression coefficient equivalent to Cohen’s *d* = 0.56, α ≤ 0.05 (one-tailed), which is aligned with systematic reviews noting moderate (~*d* = 0.60) effect sizes for parenting programs among caregivers of autistic children (19, 20). A one-tailed test was chosen based on our directional hypotheses and the wealth of evidence supporting the effectiveness of the FCU®. Given that *d* = 0.56 is somewhat larger than that found in previous studies of the FCU® in non-autistic children, we will also conduct two additional sets of analyses. First, we will calculate a Bayes Factor for each analysis to determine whether the pattern of effects are more consistent with the null (the two groups do not differ) or alternative hypotheses (the two groups differ). Second, we will conduct exploratory, descriptive analyses to determine the percentage of participants in each condition defined *a priori* as responders using the ABC and CGI, and describe intervention-, organization and policy-relevant child, family and intervention characteristics in each group.

#### 2.5.2 Implementation—aims #1–5

Quantitative data will be analyzed using a descriptive approach (e.g., counts, means, recruitment and screen-positive/negative rates, and visit attendance). This will be complemented by qualitative feedback focused on acceptability and feasibility of the research protocol.

Qualitative data will be coded by experienced research staff, supervised by experts with knowledge of the FCU® and qualitative and mixed methods. Videotaped sessions, speech samples, focus group, and interview data will be transcribed verbatim, with all transcribed and open-ended survey data collectively imported into and managed using the Lumivero (2023) *NVivo* (Version 14) www.lumivero.com platform. The research team will then apply codebook-based, thematic analysis to qualitative data sources; the codebook will be informed *a priori* by our EPIS and FCU® Implementation Frameworks, but also allow for the iterative generation of new codes and thematic domains, as data collection, analysis, and interpretation unfold (84, 85). Peer-debriefing with experts in qualitative research, the FCU®, parenting practices, and ASD will support validity of codes and determination coding sufficiency. Use of multiple coders, consensus coding approaches, interim member-checking, and thick description will ensure the integrity and reliability of our analysis.

## 3 Ethics and dissemination

The study protocol follows SPIRIT guidelines and was approved by the Hamilton Integrated Research Ethics Board (HIREB, #14475, March 2023, Version 6.0) and registered at clinicaltrials.gov (NCT05280613).

### 3.1 Informed consent

A copy of the informed consent form will be provided to the family at the screening visit (either emailed or given in person) for review and discussion prior to the baseline visit. Informed consent will be obtained by trained research staff from caregivers and children who are capable of consenting, before conduct of any study-specific procedures and after the study has been thoroughly described and all questions answered. Assent will be obtained from children who are capable of assenting. Understanding of the study will be confirmed by asking clarifying questions, e.g., “Why are we doing this study? What are some of the good/bad things that might happen in this study? Who will know what you say during the study? Do you have to take part in the study?

### 3.2 Adverse events

Adverse events (AEs) are any untoward health outcome that occurs during study participation, regardless of whether the event can be attributed to study participation. Since the FCU® is a psychological and behavioral intervention, this study involves minimal risk. Therefore, AEs will not be systematically elicited at each study contact; however if caregivers report an AE to a clinician or research staff person, it will be reported to the Research Coordinator and documented according to local Research Ethics Board guidelines. Anticipated AEs that are considered to signal unresolved risk to caregiver, child, research staff, or clinician (e.g., severe aggression or depression, suicidality) will be discussed with the PI. Child protection, police, or emergency medical service will be alerted if there is concern about imminent risk to life of an adult or safety of a child. The LPI or designated back-up person will determine if other steps must be taken to mitigate risk to caregiver, child, research staff, or clinician.

### 3.3 Risk mitigation

As the psychosocial intervention under study is considered low-risk, and delivered within a hospital-based clinical setting where clinical supervisors routinely assess safety and risk, a Data Safety and Monitoring Board (DSMB) was not assembled. Clinical concerns for both intervention and TAU participants are reviewed with a panel of clinicians and investigators and administrative leaders as issues arise. Stoppage rules were not deemed necessary for the purposes of this study.

Research staff and clinicians will be trained to evaluate and address concerns about serious or imminent mental health risk in caregivers, child safety assessments, and reporting duties, as well as crisis services available in the community. They will immediately apprise the LPI (or back-up) of concerns about child safety or serious parental mental health issues noted during study visits. Safety concerns will also be discussed at weekly team meetings supervised by a qualified psychologist and/or psychiatrist. In case of mental health or safety emergencies, the LPI or designated back-up will assess the participant and make appropriate safety and/or reporting decisions as guided by their clinical expertise and professional duties to report. All actions taken to mitigate risk and outcomes of these actions will be documented on a Risk Mitigation Report Form. If the LPI determines that a participating caregiver must be withdrawn from the study for any reason, the investigator will notify the caregivers and inform them of other available options for services in the community and, if consent is provided, notify their family physician or other health care provider of the decision. Research staff and FCU® clinicians also receive training to mitigate risk related to working with children and families. Injuries or threats to staff will be documented and discussed at weekly FCU® meetings (if occurring in FCU® intervention participants) as well as research team meetings.

### 3.4 Confidentiality, data management, and access

As part of the informed consent process, caregivers will be informed about privacy and confidentiality of data, and also about the potential need to breach confidentiality if there are concerns about any child’s safety or imminent harm to any adult necessitating advising appropriate authorities. No data will be released to third parties without the explicit written consent of the participant or their legal guardian.

Each participant will be assigned a sequential identification number and these numbers, rather than names, will be used to collect, store, and report participant data throughout the study. The study team will keep a separate log of identifiable participant information for internal tracking purposes; this log will be kept separately from data and will always be securely stored and accessible only to research staff. Research study source documents will be kept securely in password-protected files on secure McMaster servers, or in locked storage at the Offord Centre for Child Studies. Paper and electronic data will be stored securely for a minimum of 7 years after final study report or primary peer review publications. All staff will be PHIPPA trained.

Source documents are defined as original documents, data, and records. They may include hospital records, clinical and/or office charts, clinical notes or evaluation checklists, videotaped observations, and communication records (e.g., telephone logs, emails). Study staff will clearly define the various source documents used to support the study as part of their local data management processes. Data collection will be completed by authorized study site personnel designated by the LPI. Participants will not be identified in the study database by name or initials; they will be identified by their unique participant ID.

Survey data will be collected on password-protected laptops and tablets and de-identified at the point of collection. Where possible, questionnaire data will be collected in electronic format using Qualtrics, an Application Service Provider (ASP) using a Software-as-a-Service (SaaS) platform for creating and distributing online surveys and other research services. Qualtrics uses Transport Layer Security (TLS) encryption for all transmitted internet data. Its services are hosted by trusted third party data centers that are audited using the industry standard SSAE-16 SOC 1 Type 2 method. All data are stored within the region where data are collected and will not be moved from that region.

### 3.5 Data processing and data management

Data will be checked for valid values and ranges, between item logical consistency, and within-participant variation. Participants will be included in the data analysis provided they complete all screening and baseline procedures, and that there is at least some post-baseline data available. All study-related source data will be entered into the study database. Only FCU® intervention data listed as study-relevant variables will be directly entered into the database (e.g., outcomes, covariates, mediators, moderators, and exploratory variables). If items are left blank when these measures are completed, the standard procedure as outlined in the manual for each questionnaire will be followed to account for missing data. Site staff will ensure that the study records for all participants are up to date as soon as possible soon after participant completion of study, with field and form exceptions reviewed and accepted to account for all required data.

### 3.6 Dissemination

We will integrate implementation and effectiveness findings into renewed quality improvement and adaptation of the FCU® model. Should the proof-of-principle effectiveness study fail to demonstrate significant intervention effects, we will integrate qualitative and quantitative findings to enhance the model and/or target population and re-evaluate within a larger RCT or pre/post study. We will publish findings in academic journals; clinical, family, and academic conferences and presentations.

## 4 Conclusion

Everyday relationships, activities, and interactions, including positive parenting and cohesive family relationships, represent key mental health protective factors throughout the development of autistic children. Such protective factors are themselves shaped by the broader social determinants of child and family health, including household income security and social supports. Autistic children and youth who experience co-occurring EBP are more likely to experience psychosocial adversity, such as caregiver depression, income stress, and social isolation. These risk factors can compound negative effects by acting as barriers for families seeking to access autism services, parenting support, and mental healthcare. While positive parenting approaches have demonstrated robust and reliable effects on child EBP in families of autistic children, there is a paucity of intervention approaches that effectively address the developmental, mental health, and psychosocial complexity present in the daily lives of many autistic children and their families.

The study was designed as a response to research that suggests that treatment will have greatest chance of success if it: (a) provides a comprehensive assessment of the social-ecological influences on child and family, (b) effectively engages and supports caregivers dealing with greater life complexity and psychosocial strain, (c) efficiently tailors family-centered care plans, and (d) targets positive parenting practice as a path to shaping child self-regulation. Furthermore, research-to-practice gaps often mean long lags and low uptake of evidence-based interventions into regular clinical care. Hybrid implementation-evaluation approaches seek to shorten this gap by evaluating the effectiveness of interventions within real-world settings while simultaneously describing barriers and facilitators related to program uptake, sustained delivery and scale-up.

In the current study, we will evaluate the FCU®, an assessment-driven ecologically sensitive model of family-centered care aimed at preventing and/or decreasing EBP in children and supporting positive parenting and family well-being in children at high risk of persistent EBP, within a “real-world” autism service setting. The study has several strengths with respect to intervention and study design: Several features of the FCU® enhance its fit for families and children experiencing developmental, mental health, and psychosocial complexity, including a strength-based focus, positive parenting lens, motivational approach, and an emphasis on tailoring intervention to diverse child and family needs. The study design includes both caregiver report, blinded independent evaluator-led interviews, and observational measures. The mixed methods RCT design will enable a more comprehensive and nuanced understanding of caregivers’ experience of the program and processes of change aimed at explaining varied patterns of response (and non-response) to intervention and will enable qualitative comparison and triangulation of data between participating caregivers and FCU® providers. Finally our team is composed of clinicians, administrators and researchers, which has facilitated delivery of the RCT within an established autism service setting with commitments to sustain and/or adapt delivery of the model for further research or quality improvement as needed. By embedding the study in a real-world setting, we hope to create a more efficient research-to-practice pathway.

We anticipate encountering limitations in the study, particularly in relation to trade-offs inherent in more pragmatic designs. First, our research question investigates whether intervention participation in the FCU® confers advantages to autistic children and youth referred for care within an established clinical setting. Because this question is of interest to administrators and clinicians, we elected to include a broad sample of clients/patients with respect to age and cognitive and spoken language ability. Second, differential response to caregiver-led intervention may occur based on child or caregiver characteristics (e.g., child co-occurring ID) however our sample size is not powered to detect moderator effects. We will provide preliminary descriptive estimates of effect sizes by child age and ID status as stratification factors. Finally, using an effectiveness, rather than efficacy, design, we did not validate diagnoses with gold standard tests such as the Autism Diagnostic Interview-Revised (ADI-R) (86) and/or Autism Diagnostic Observational Schedule (ADOS-2) (87). It is possible that some clients will be referred to an autism service whose profile is best understood by other diagnoses than autism, however this compromise was made to facilitate clinical flow and assess the added value of the FCU® to clinic clients “as is.” Using the SCQ-C (67), we will explore whether autistic symptom severity is associated with indices of response to intervention.

Scalable, ecologically focused family-centered interventions offer promise as key components of a public health framework aimed at reducing mental health inequities among autistic children and youth, and their caregivers. Results from this study will inform further adaptations and evaluation efforts aimed at “making the race fair” for autistic children and youth and their families.

## Ethics statement

The studies involving humans were approved by Hamilton Integrated Review Board (HiREB), McMaster University. The studies were conducted in accordance with the local legislation and institutional requirements. Written informed consent for participation in this study was provided by the participants’ legal guardians/next of kin in instances where participating children and youth were deemed unable to provide consent themselves.

## Author contributions

TB: Conceptualization, Funding acquisition, Investigation, Methodology, Project administration, Resources, Supervision, Visualization, Writing – original draft, Writing – review & editing. ID: Conceptualization, Investigation, Methodology, Supervision, Writing – original draft, Writing – review & editing. JG: Conceptualization, Project administration, Writing – original draft, Writing – review & editing. MJam: Formal analysis, Methodology, Writing – review & editing. MK: Conceptualization, Methodology, Writing – review & editing. AZ-Z: Conceptualization, Methodology, Writing – review & editing. KA: Conceptualization, Methodology, Resources, Writing – review & editing. JF: Supervision, Writing – review & editing. ED: Formal Analysis, Methodology, Writing – review & editing. SG: Conceptualization, Methodology, Visualization, Writing – review & editing. AG: Conceptualization, Methodology, Resources, Visualization, Writing – review & editing. MJan: Conceptualization, Writing – review & editing. EL: Writing – review & editing. PP: Conceptualization, Supervision, Writing – review & editing. HP: Conceptualization, Methodology, Writing – review & editing. CR: Resources, Supervision, Writing – review & editing. MS: Methodology, Writing – review & editing. RS: Supervision, Writing – review & editing.

## In memoriam

This study is dedicated to the late Dr. Thomas Dishion, with gratitude.
